# Nanomechanics of multidomain neuronal cell adhesion protein contactin revealed by single molecule AFM and SMD

**DOI:** 10.1038/s41598-017-09482-w

**Published:** 2017-08-18

**Authors:** Karolina Mikulska-Ruminska, Andrej J. Kulik, Carine Benadiba, Ivet Bahar, Giovanni Dietler, Wieslaw Nowak

**Affiliations:** 1 Laboratory of Physics of Living Matter, Ecole Polytechnique Fédérale de Lausanne (EPFL), CH-1015 Lausanne, Switzerland; 2 Institute of Physics, Faculty of Physics, Astronomy and Applied Informatics, Nicolaus Copernicus University, Grudziadzka 5, 87-100 Torun, Poland; 3 Department of Computational and Systems Biology, School of Medicine, University of Pittsburgh, 3501 Fifth Ave, Biomedical Science Tower 3, Pittsburgh, PA 15213 USA

## Abstract

Contactin-4 (CNTN4) is a complex cell adhesion molecule (CAM) localized at neuronal membranes, playing a key role in maintaining the mechanical integrity and signaling properties of the synapse. CNTN4 consists of six immunoglobulin C2 type (IgC2) domains and four fibronectin type III (FnIII) domains that are shared with many other CAMs. Mutations in CNTN4 gene have been linked to various psychiatric disorders. Toward elucidating the response of this modular protein to mechanical stress, we studied its force-induced unfolding using single molecule atomic force microscopy (smAFM) and steered molecular dynamics (SMD) simulations. Extensive smAFM and SMD data both indicate the distinctive mechanical behavior of the two types of modules distinguished by unique force-extension signatures. The data also reveal the heterogeneity of the response of the individual FNIII and IgC2 modules, which presumably plays a role in the adaptability of CNTN4 to maintaining cell-cell communication and adhesion properties under different conditions. Results show that extensive sampling of force spectra, facilitated by robot-enhanced AFM, can help reveal the existence of weak stabilizing interactions between the domains of multidomain proteins, and provide insights into the nanomechanics of such multidomain or heteromeric proteins.

## Introduction

**Figure 1:**
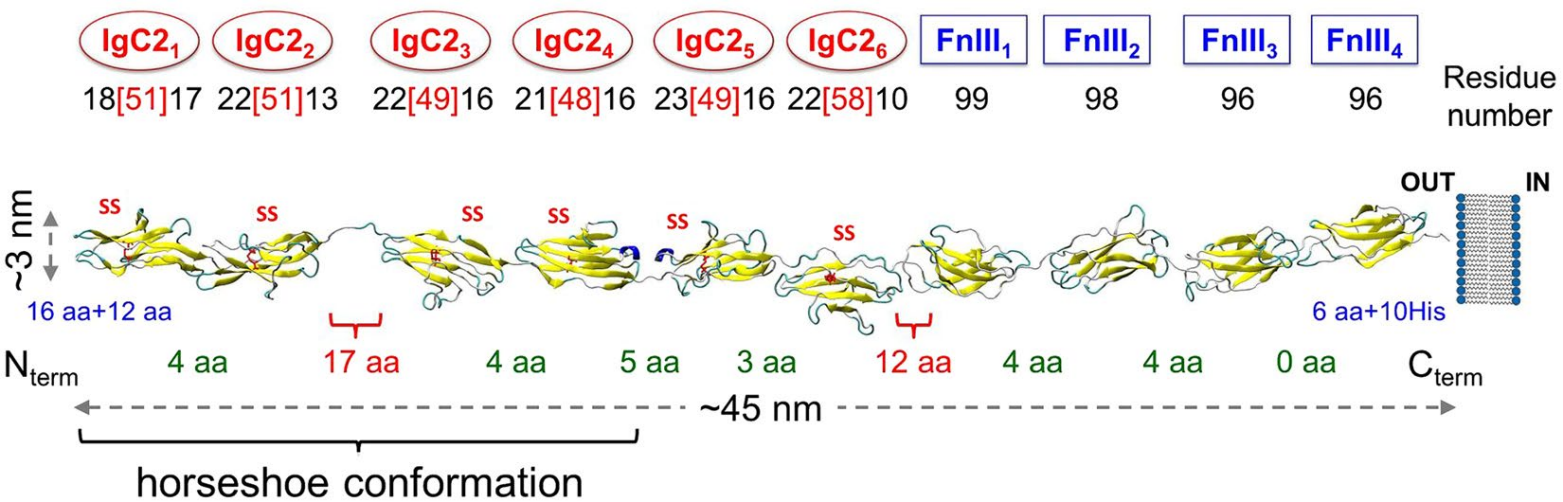
Modular structure of CNTN4. The protein consists of six N-terminal IgC2 domains and four fibronectin type III (FnIII) domains. Each IgC2 domain is restrained by one disulfide bond (SS) that protects half of the domain (marked by red licorice). The numbers of residues that form each domain have been described in the following way: total number of residues for FnIII modules and split into three parts for IgC2 modules i.e. the number of residues harbored by disulfide bridge (in the square bracket) surrounded by the number of residues before and after disulfide bridge. The length of the protein is ~45 nm, when the domains are aligned. The number of free amino acids (aa) linking each pair of consecutive modules is shown. The first four IgC2 domains preferentially assume a horseshoe conformation.

Cell migration, axonal growth and cell adhesion are fundamental processes in the development of the nervous system^1^. There is growing evidence in support of the significance of mechanical driving forces and interactions for regulating these fundamental processes, in addition to the well-established biochemical and genetic bases of their function^2,3^. Adhesive contacts between neuronal cells are essential to establishing and maintaining the proper architecture, connectivity, plasticity, and thereby function, of neurons in the central nervous system (CNS). These contacts are enabled by cell adhesion molecules (CAMs) such as contactins (CNTNs). CNTNs are neuronal membrane proteins that belong to a subgroup of the immunoglobulin (Ig) superfamily of CAMs (IgCAMs) associated with the brain^4^. They are involved in axonal growth, guidance and fasciculation^5^, in addition to linking pre- and postsynaptic cells. Six forms of CNTNs (CNTN1-CNTN6) have been discovered so far. Recent study indicated the involvement of CNTN1 in promoting cancer metastasis was reported^6^. We focus here on contactin-4 (CNTN4), also known as BIG-2 (Fig. 1). CNTN4 is present in various regions of the brain (e.g. the hippocampus, nuclei of the thalamus, cerebral neocortex, olfactory bulb and substantia nigra) consistent with its role in maintaining neuronal networks and CNS development^7,8^. CNTN4s are tethered by a glycosylphosphatidylinositol (GPI)-linker to the pre-synaptic membrane^9,10^; and have several binding partners such as the amyloid precursor protein (APP), secreted APP ectodomain (APPsα), amyloid-like protein 1 (APLP1), and protein tyrosine phosphatase receptor γ (PTPRγ). In spite of their vital biological role, the properties and interactions of CNTNs at molecular level remain to be established^9^.

All CNTNs share a modular structure characterized by six N-terminal Ig domains type C2 (IgC2_1_- IgC2_6_) and four fibronectin type III domains (FnIII_1_-FnIII_4_), in addition to their C-terminal GPI-anchoring domain attached to the synaptic cell membrane extracellularly^11^. They are composed of over 1,000 residues and may extend up to a length of ~45 nm, hence their ability to span the cleft of ~ 30–60 nm between pre- and post-synaptic cells, and link myelin to axon membrane. While the structures of their individual domains of similar types are generally known, their intact structure under physiological conformation remains known. CNTN4 interacts with PTPRγ through its second and third Ig domains. During this interaction, CNTN4 adopts a horseshoe like conformation, observed also in the CNTN2^12^ and other IgCAMs such as hemolin, axonin, Dscam and neurofascin^13^. Protein kinase, protein-tyrosine phosphatase and cytokine receptor families also share similar domains with CNTNs.

Mechanical effects at multiple time and length scales are implicated in a diversity of neurobiological processes, ranging from molecular events (e.g. mechanosensitive channels, CAMs, actin filaments), to tension-dependent exo- and endocytosis, synapse formation, neuronal migration and axonal growth, to neural circuit formation^2^. In particular, adhesion-dependent cell mechanosensitivity is a fundamental process of broad interest probed by approaches that measure local forces at adhesion sites^14^. Advances in single molecule force spectroscopy now permit us to measure forces of the order of piconewtons (pN). A seminal example is the force-dependent recruitment of vinculin to focal adhesion proteins, which draws attention to the significance of the dynamics of cell-adhesion complexes. Understanding the response of load-bearing structures (such as CAMs) to mechanical stress is essential to gaining insights into the mechanism of transduction of forces into biochemical signals^15^. Yet, the nanomechanics of CAMs remains to be established and this is a field of vigorous research^16–18^.

Toward elucidating the nanomechanics of CNTN4 – a representative neuronal CAM, we used atomic force microscopy (AFM) to record single molecule force spectra associated with its mechanical unfolding. Single molecule force spectroscopy (smFS) experiments characterize the mechanical response of biopolymers at the nanometer scale^19^. In this report, we show that smFS successfully provides information on not only the forces (or strains) associated with mechanical unfolding of individual CNTN4 domains but also the strengths of interdomain interactions. It is known that smFS alone cannot reveal atomic details on conformational changes accompanying mechanical stresses, especially for large and complex systems. This information is obtained with the help of Steered Molecular Dynamics (SMD) simulations adopted here for the first time for a full length CNTN. SMD has been established as a useful auxiliary tool for interpreting smFS results^20–22^, for example in titin stretching.

In the present study, we report for the first time the smAFM spectra for fully elongated CNTN4 structure (up to 10 unfolding peaks) and provide accurate estimates for the *unfolding lengths* of the different domains. Unfolding length refers to the change in the distance between the N- and C-termini of the FnIII domain induced by uniaxial tension, or to the elongation of IgC2 type domain upon partial unfolding. This is a significant advance over earlier studies^23–26^, both in terms of the statistical accuracy (>2,800 unfolding domain peaks observed here, as opposed to <300 in earlier work^28^) and size of the system (intact full-length multidomain CNTN with more than 1,000 residues) whose mechanical behavior has been characterized. Our study further highlights and explains characteristic features of CNTN4 unfolding spectra with the help of two computational approaches - SMD (similar to earlier work^27–29^) and mechanical stiffness (*MechStiff*)^30,31^ analysis based on the anisotropic network model^32^. *MechStiff* permitted us to estimate the mechanical strength of the individual IgC2 and FnIII domains, which are similar in their folds but distinct in their amino-acid composition.

## Results and Discussion

### IgC2 and FnIII domains exhibit distinctive mechanical responses detected by smFS

**Figure 2:**
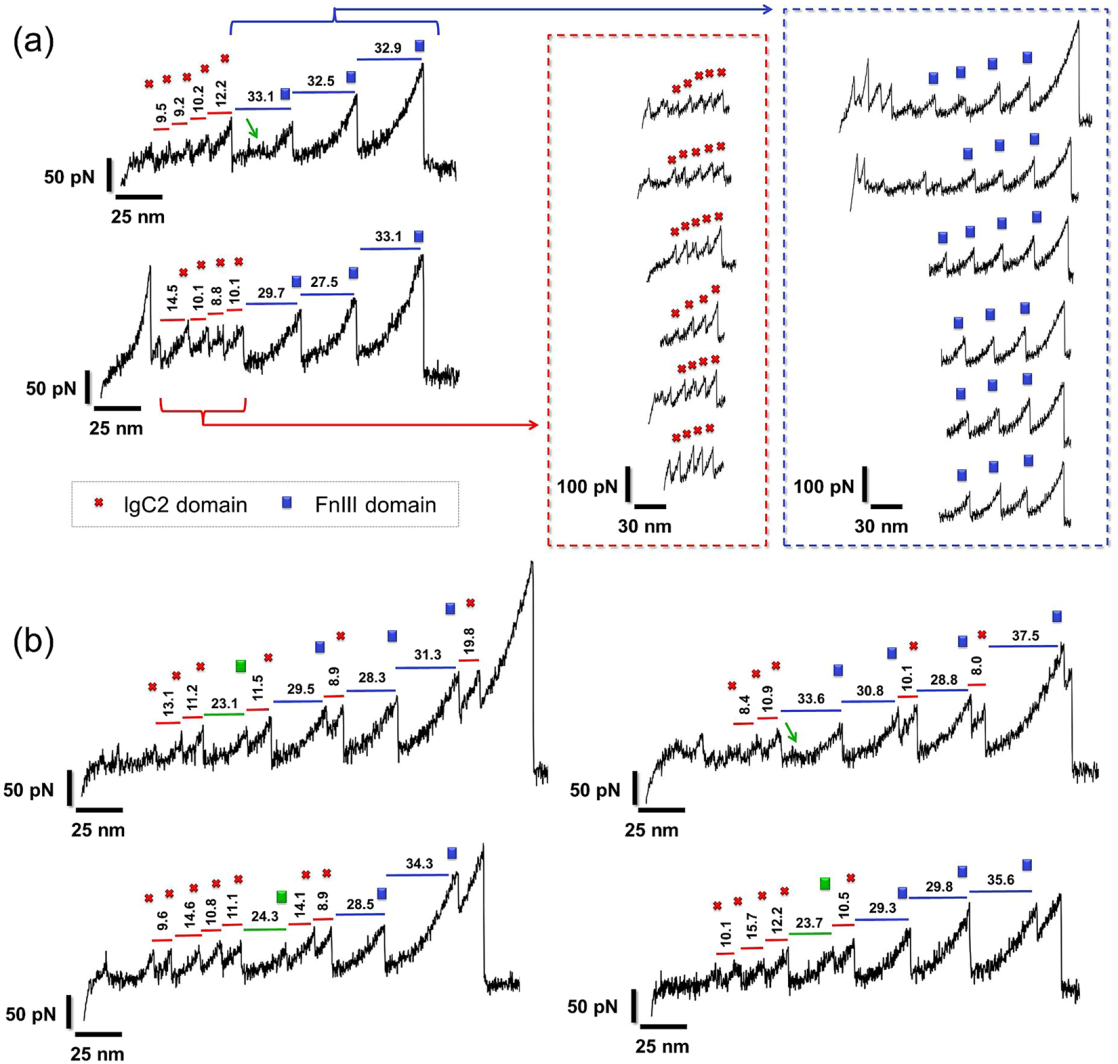
Typical force-extension curves with saw-tooth patterns observed for CNTN4 in smFS AFM. Red crosses and blue rectangles indicate unfolding patterns associated with the respective domains IgC2 and FnIII. Displayed numbers (in nm) above each peak are the unfolding length resulting from WLC model fitting. Green arrow points a characteristic hump which appears in FnIII modules. (**a**) Examples of force-extension profiles with sequential unfolding of short (red crosses) and long (blue rectangles) elongations are displayed in red and blue boxes (right side); (**b**) Example of non-sequential unfolding of short and long elongations with at least 9 unfolding peaks. Green square indicates an event that most probably arises from alternative unfolding of FnIII1 module.

The stress-strain (or force-extension) curves obtained by smAFM experiments for CNTN4 exhibit a saw-tooth pattern where each tooth corresponds to the unfolding of a domain or its part (Fig. 2). IgC2 and FnIII domains contain similar numbers of residues; but IgC2s also comprise disulfide bonds that confer additional resistance to mechanical stress and result in smaller contributions to unfolding length (Fig. 1). The presence of one disulfide bond in each IgC2 module presents a force clamp, and thus partial unfolding originating from such a module are possible. The substantial part of the IgC2 chain is sheltered from extension via this Cys-Cys bridge. In force-extension curves, we distinguish two types of unfolding peaks (Fig. 2a; *red crosses* denote IgC2 domains and *blue rectangles*, the FnIII domains).

**Figure 3:**
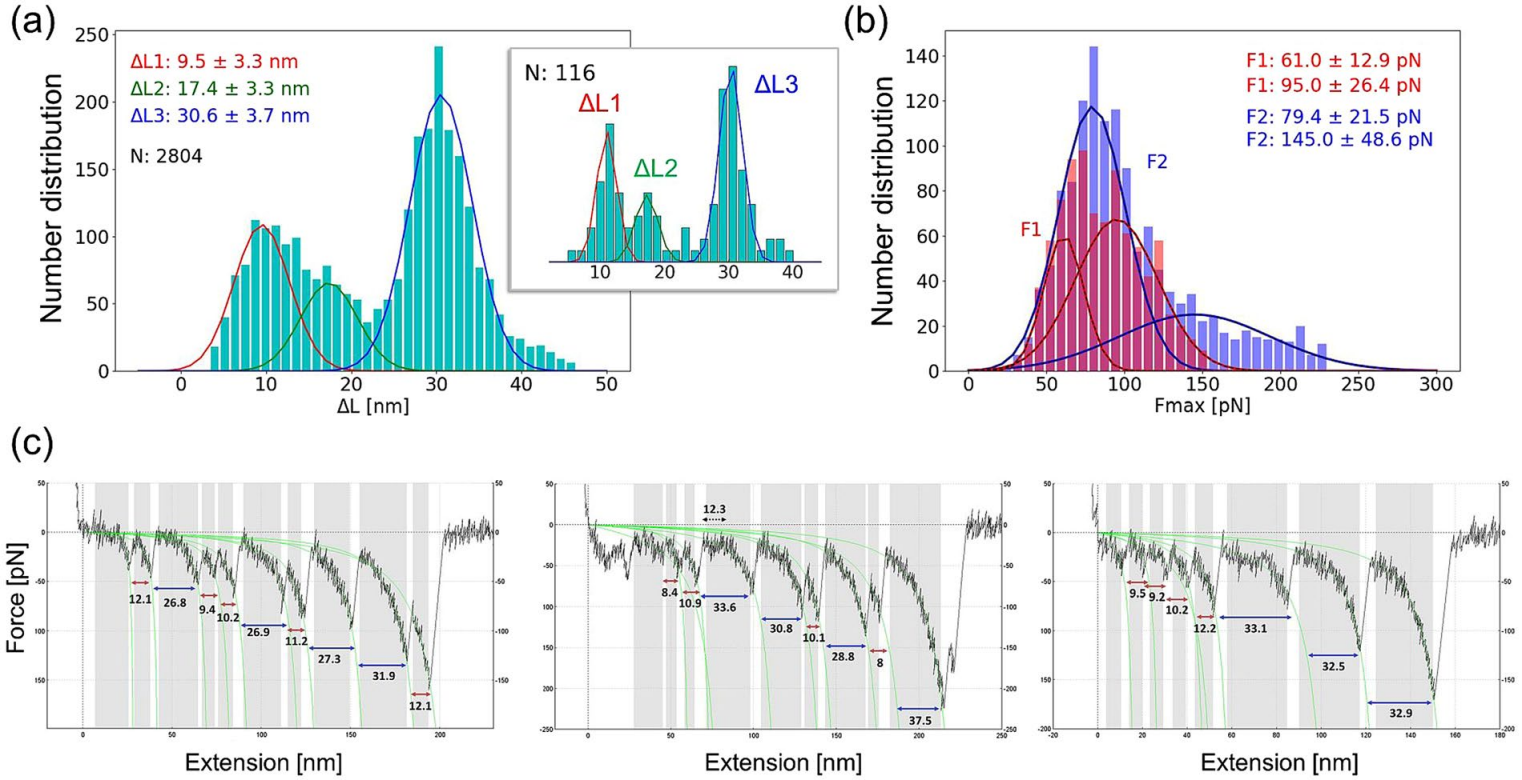
Distribution of unfolding lengths (ΔL, in nm) and maximal forces obtained from WLC fitting of experimental data. (**a**) Results from fitting a total of 2,804 unfolding by two Gaussian functions with respective mean values of ΔL = 9.5 ± 3.3 nm and 17.4 ± 3.3 nm (corresponding to IgC2 domains) and 30.6 ± 3.7 nm (FnIII domains). The inset displays the histogram obtained from 21 most representative unfolding curves (116 peaks), which indicates similar results: ΔL_1_: 11.0 ± 1.6 nm, ΔL_2_ ≈ 17.2 ± 1.8 nm and ΔL_3_: 30.4 ± 1.8 nm. (**b**) The distribution of maximal forces (F_max_, in pN) obtained from WLC model. Data for IgC2 domains (*red*) are fit by three Gaussian distributions, with mean values: 61.0 ± 12.9 and 95.0 ± 26.4 pN; and those for FnIII, by two Gaussians with means 79.4 ± 21.5 and 145.0 ± 48.6 pN. (**c**) Example of force-extension curves with WLC fitting.

In order to estimate the change, ΔL, in the end-to-end distance (or unfolding length) of each module and the corresponding maximal force (F_max_), we used the WLC model^33^. Figure 3 displays the histograms of ΔL based on careful selection of over 500 force-extension curves (examples are shown in Fig. 3c) that exhibited a total of 2,804 peaks in the course of stress-induced unfolding. Three characteristic lengths are obtained by Gaussian fits to the histograms: ΔL_1_ = (9.5 ± 3.3) nm, ΔL_2_ = (17.4 ± 3.3) nm and ΔL_3_ = (30.6 ± 3.7) nm. The most probable extension (ΔL_3_, highest peak in Fig. 3a) corresponds to FnIII domains, and the lower two, to IgC2 domains. This interpretation is supported by classical estimates of the unfolding lengths of IgC2 modules (see Table S1 in SI).

We further analyzed the subset of data that exhibited a strong attachment to the smAFM piezoelectric platform (distinguished by high stress at the final stage of the experiment), which also displayed 7 to 10 peaks. This subset yielded similar results, with ΔL_1_: 11.0 ± 1.6 nm, ΔL_2_ ≈ 17.2 ± 1.8 nm and ΔL_3_: 30.4 ± 1.8 nm (Fig. 3a, small histogram in the inset).

### CNTN4 smAFM Spectra Reveal Force-Extension Patterns Characteristic of Specific Conformational Changes

**Figure 4:**
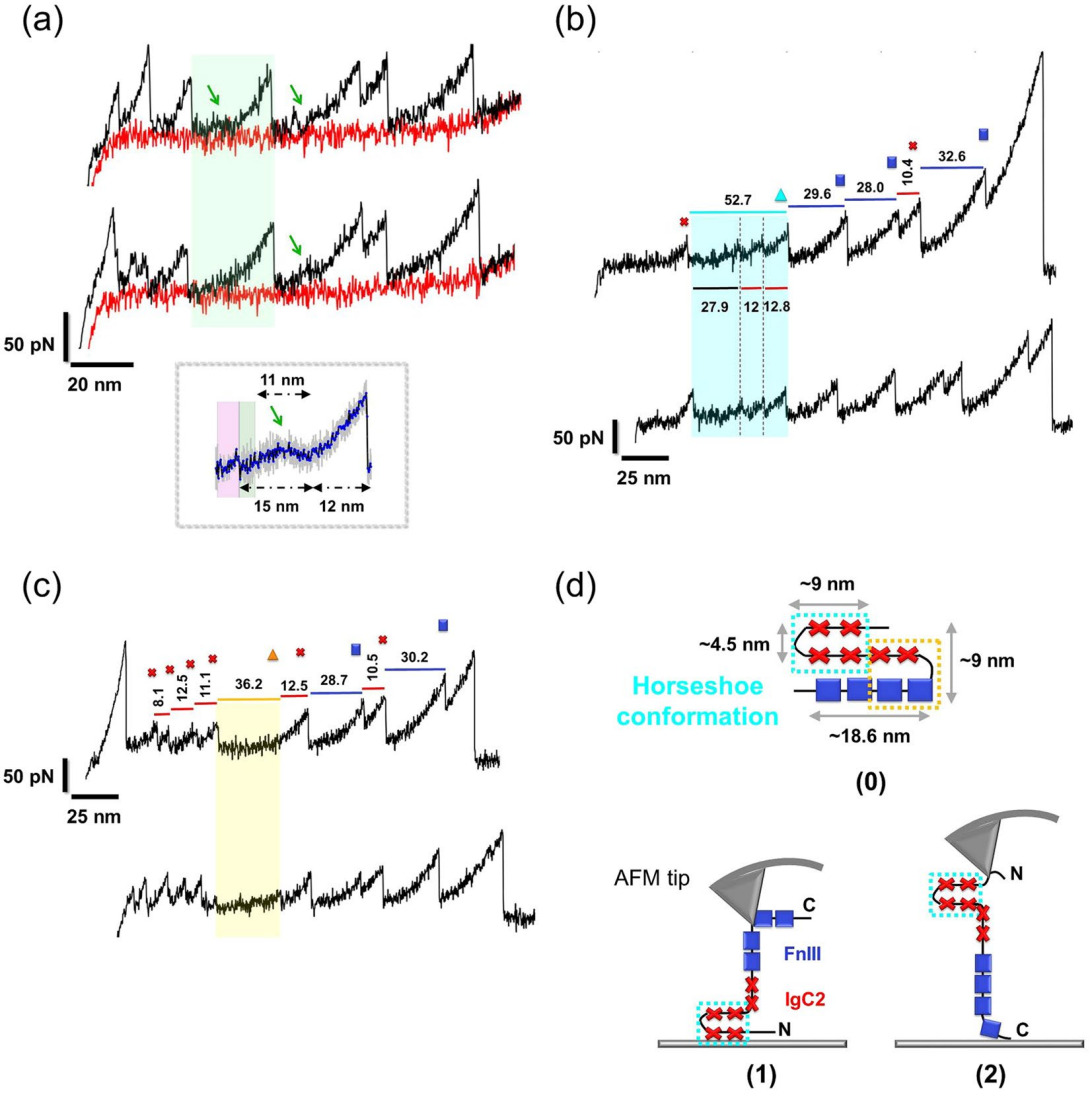
Force-extension curves with sawtooth patterns and characteristic of specific conformational changes observed for CNTN4 in smFS AFM. (**a**) Force-extension curves with similar unfolding scenarios. Green arrows show smoothly raising “plateau” with a clear ~13–18 nm hump, potential intermediate state in FnIII domain. Green area highlights similar unfolding event of FNIII module with (top curve) and without (bottom curve) the hump. In the inset the average of 7 similar unfolding peaks (blue curve) with standard deviation (grey) is shown. Panels (**b**) and (**c**) present long “plateaus” (blue, yellow areas) proceeded by a partial unfolding of a single (**b**) or several (**c**) Ig domains. (**d**) Hypothetical structure of adsorbed native CNTN4 with a horseshoe conformation (light-blue box) (0), possible unfolding arrangements of CNTN4 with the horseshoe fold of IgC2_1–4_ modules are shown in schemes (1) and (2).

The smAFM spectra of native large multimodular proteins are challenging to interpret due to possible artefacts induced by complex mechanical unfolding scenarios and compact structures of proteins adsorbed on a glass surface. However, due to a large body of AFM data, we have detected representative patterns presented in Figs 2b and 4.

(I) An event frequently registered in the smFS spectra of CNTN4 was the occurrence of approximately 30 nm long, smoothly rising “plateau” with a ~13–18 nm hump (see Figs 2a and 4a, *green arrow*). This feature probably represents the passage over an intermediate state during the unfolding of FnIII module, which would repeat multiple times on the same smFS curve (Fig. 4a, average of 7 recording shown in the inset) or would not appear at all in the spectrum (Fig. 4a, lower curve). It is known that complex protein structures may select from several unfolding pathways, and occasionally visit intermediate states. Therefore, only in some experiments does this type of hump appear. This scenario is presented in Fig. 4a where we have similar unfolding curves and we can see just one unfolding peak of FnIII module with and without the intermediate state at ~11 nm.
(II) We observed an unfolding length of 23.1–24.4 nm (WLC estimate) in the initial phase of unfolding (see Fig. 2b, *green square*). It doesn’t fit to typical FnIII nor IgC2 modules lengths. A simple estimate based on 0.36–0.4 nm persistence length per one amino acid indicates that in this event some 50–55 amino acids should be involved. The plausible explanation of this feature is an assumption that partial unfolding of one FnIII domain occurs. Similar unfolding of length ~20 nm was observed by A.F. Oberhauser et. for the FnIII_1_ module of fibronectin^34^. Based on FNIII domain lengths we infer that partially unfolded FNIII_1_ module of CNTN4 is responsible for this feature. Moreover, the discussed intermediate unfolding length always appears after IgC2 domains unfolding and at most three FnIII unfolding peaks are observed in the same spectrum.
(III) Another feature is characterized by plateaus having extension lengths of 35–45 nm. Assuming that these are not systematic artefacts, these plateaus, due to their lengths, do not correspond to any obvious conformational rearrangements of CNTN4. The features are not completely flat and they appear during the unfolding of IgC2 domains with some additional initial length (see Fig. 4b,c, regions marked by blue and orange triangles). Notably, this signal appears only before the unfolding of FnIII domains.

It is tempting to propose that this feature may result from a reorganization of the IgC2 domains, from a horseshoe like conformation, observed in X-ray structure^12^ to an extended one upon deformation of the three or four IgC2 domains, as shown in Fig. 4d. Sometimes we observed those features at early stages of CNTN4 unfolding, where only one IgC2 peak precedes the plateau and two IgC2 modules unfold afterwards (Fig. 4b, blue triangle). In that case a hypothetical scenario (1) or (2) from Fig. 4d might fit to those smAFM spectra. In another arrangement of CNTN4 on the AFM tip the initial few peaks would correspond to unfolding of IgC2_1_ - IgC2_4_ domains (Fig. 4c) and releasing of Ig-FnIII interaction (Fig. 4d, yellow box). However, we cannot exclude that such plateaus result from less specific deformations of CNTN4. We measured shapes and sizes of CNTN4 adsorbed on glass and graphene (data not shown) and have found that circular shapes with about 10 nm radius prevail in flat (~3 nm) isolated spots detected in the AFM tapping mode imaging.

The force-extension curves obtained by smAFM also provide information on the maximal forces (F_max_) attained before triggering the successive unfolding events. Recorded data have been sorted using the unfolding length of CNTN (Fig. 3b) as a reference. The F_max_ values for FnIII domains are best fit by two Gaussian distributions, with mean values of 79.4 ± 21.5 pN and 145.0 ± 48.6 pN, comparable to those measured for fibronectin (FN)^34,35^: some FnIII modules of CNTN4 have mechanical strengths similar to those of FnIII_10_ and FnIII_13_ (~70–90 ± 20 pN), and others are stronger than FnIII_12_ and reach up to 125 ± 20 pN^34^. Moreover, contrary to available literature data^34,36,37^, in CNTN4 we have found lower unfolding force for IgC2 domains than for FNIII domains, namely 61.0 ± 12.9 vs 95.0 ± 26.4 pN. The broad distribution is attributed to the different sizes and sequences of the six IgC2 modules. Similar distributions were observed in the SMD unfolding of individual IgC2 modules of CNTN4^25^. Interestingly, our average unfolding force for CNTN4 IgC2 domains is 2–3 times smaller than that corresponding to the Ig modules of titin^34^. This difference is expected since the disulfide bridge clamp allows for much smaller unfolding lengths of Ig modules in CNTN4 in comparison to titin.

To date, two smAFM studies have examined the mechanical unfolding of CNTN4^23,24^. The experimental conditions therein were different than those adopted in the present study; and statistically limited data on CNTN4 unfolding events were collected at low resolution of forces. The first, by Dabrowska *et al*., reported data on less than 300 unfolding events^23^. The distribution of unfolding lengths showed maxima at 18.6 ± 5.1 nm (attributed to IgC2 domains) and 29.2 ± 6.7 nm (FnIII domains). The results showed also that FnIII domains required about 100 pN to unfold with the loading rate (r_f_) of 10–30 nN/m. However, the authors did not observe the unfolding peaks of IgC2 modules without TCEP (Tris(2-carboxyethyl) phosphine hydrochloride) treatment i.e. without cutting the disulfide bonds. In the second study^24^, the force distribution from less than 200 unfolding events was analyzed using the WLC model^33^. Three values of unfolding lengths were determined: 19.4 ± 10.6, 24.5 ± 3.6 and 36.8 ± 9.8 nm. The two larger values were linked to data reported earlier and the unfolding length of 19.4 nm was attributed to the presence of partially unfolded IgC2 domains.

To our knowledge, this is the first AFM smFS measurement which shows that IgC2 maximal extension is achieved by forces (F_max_) that are weaker than, or at most equally strong as, those required to achieve full extension of the FnIII modules in the same protein. However, we also note that the deformation of the IgC2 modules is almost 1/3 of that of FnIII. As a result, in terms of stiffness (i.e. the ratio of force to elongation), IgC2 is stronger than FnIII, despite requiring smaller force to extend its portion that is not protected by the disulfide bridge. The different flexibilities of the Ig and FnIII modules in some CAMs, like CNTN4, and the horseshoe-like IgC2_2_-IgC2_3_ conformation, may be essential to their function.

The heterogeneous modular structure of CNTN4 (comprised of two types of modules with distinctive mechanical characteristics) may assist in the adaptability of the structure to different widths of the synapses, and their fluctuations. CNTNs interact with various membrane proteins and extracellular matrix components while maintaining cell-cell communication/adhesion properties in axons and the chemical synapses. It is tempting to speculate that, for example, they buffer the influence of mechanical fluctuations present in the synaptic cleft to protect other proteins’ fragments against mechanical stress in a protein complex. It has been shown that CNTN1 often forms a complex with other CAMs, contributing to the extension of oligodendrocytes’ membranes to initiate myelin formation^38,39^. CNTN4 may play a similar role in other area of the CNS. It is also important to note that the IgC2 module is partly protected from stress-induced unfolding due to a central disulfide bond which is presumably responsible for maintaining IgC2 stability, while flexible parts ensure adaptability to intermolecular interactions and cell-adhesion function.

### Stress-Induced Unfolding *in Silico* Highlights the Heterogeneity of the Mechanical Response

Constant velocity SMD was used to study CNTN4 structural changes in response to tension applied along its N-to-C direction. During SMD simulations, a much faster extension rate is applied, compared to experiments^40^. Slower simulations might be more realistic but they would require prohibitively expensive computing time, especially for multimodular structures of more than 1,000 amino acids (like CNTN4). On the other hand, a fast SMD unfolding may introduce artifacts into trajectories. We made an effort to maintain the optimal balance between computational efficiency and accuracy in this study. We adopted in our SMD runs, a constant pulling speed *v* that was six orders of magnitude faster than that taking place in AFM experiments, and our spring constant *k in silico* was one order of magnitude larger than those in AFM. It has been observed that *k* affects the nature of unfolding force profile. However, there is still no precise recommendation for the ideal spring constant^41^. The product of these two parameters is known as the *loading rate r*_*f*_* = k v*. The force acting on the protein in our study was larger than that in experiments by a factor of 40. Yet, even such simulations where the expected effects are significantly accelerated provide insights into possible conformational changes during unfolding events, or help elucidate the origins of force clamps for interpreting AFM data^42–46^.

**Figure 5:**
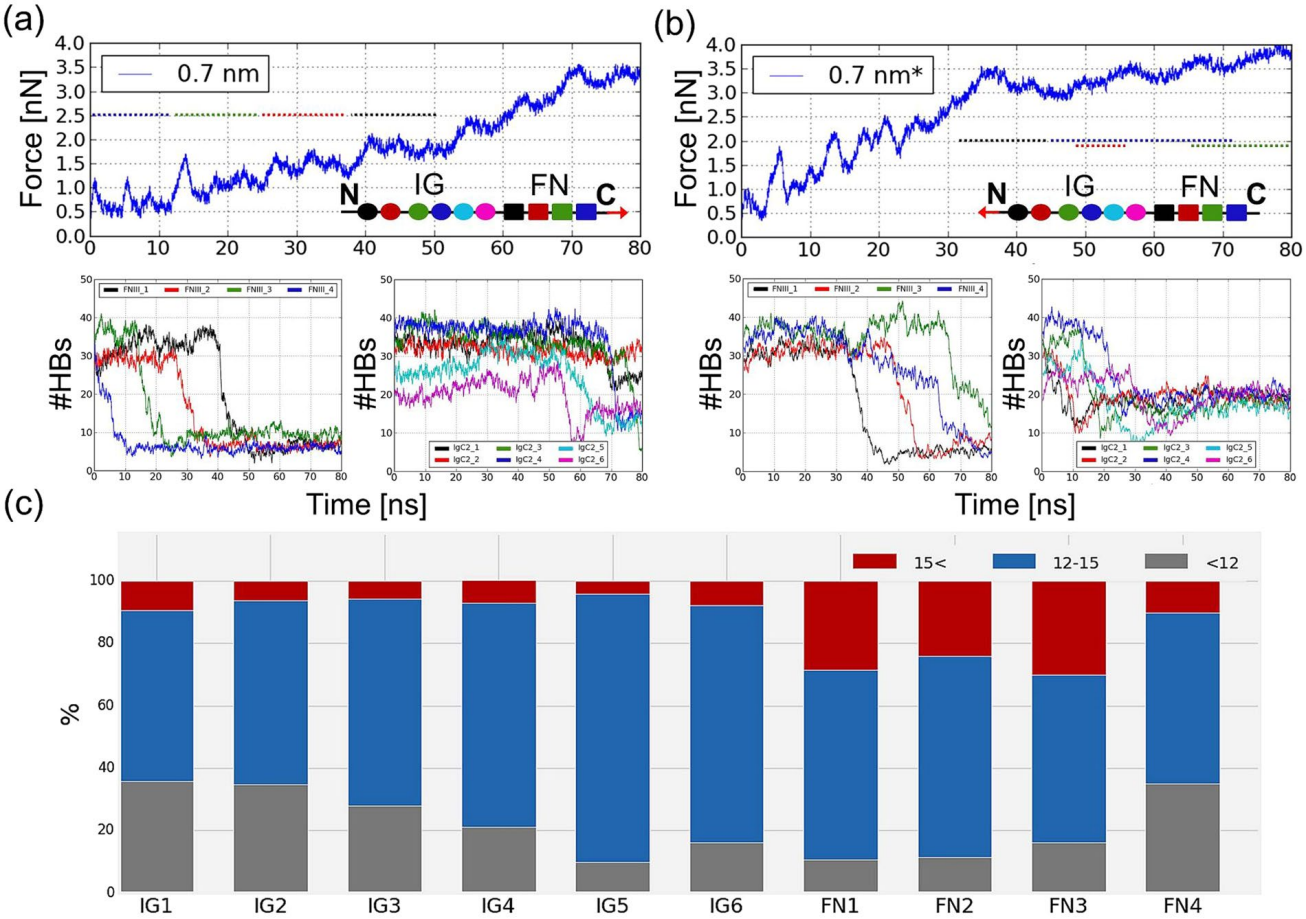
Results from SMD simulations of CNTN4. (**a,b**) For each simulation the time evolution of the experienced force under constant-velocity (0.025 Å/ps) pulling *in silico* experiments is shown (*top* curves). On the *botom*, the time evolutions of the number of hydrogen bonds (#HBs) for the FnIII_1–4_ and IgC2_1–6_ domains are displayed. Simulations were performed in the presence of a water layer of thickness 0.7 nm. The modular structure of CNTN4 in the inset shows the direction of pulling (or the application of force, either to the N-terminus or to the C-terminus, indicated by the red arrow). The colors encode the corresponding CNTN4 modules. The dotted lines in the inset show the time period during which the secondary structure melting of an encoded FnIII domain took place. (**c**) Results from *MechStiff* calculations. The results provide information about the composition of effective spring constant (*k*) in the presence of external force for each CNTN4 module, grouped in three ranges: k < 12 (*gray*), 12 ≤ k < 15 (*blue*) and k ≥ 15 (*red*), corresponding to the softest, moderate and stiffest responses, respectively. For IgC2 domains only the part which is not protected by a disulfide bond was considered.

**Figure 6:**
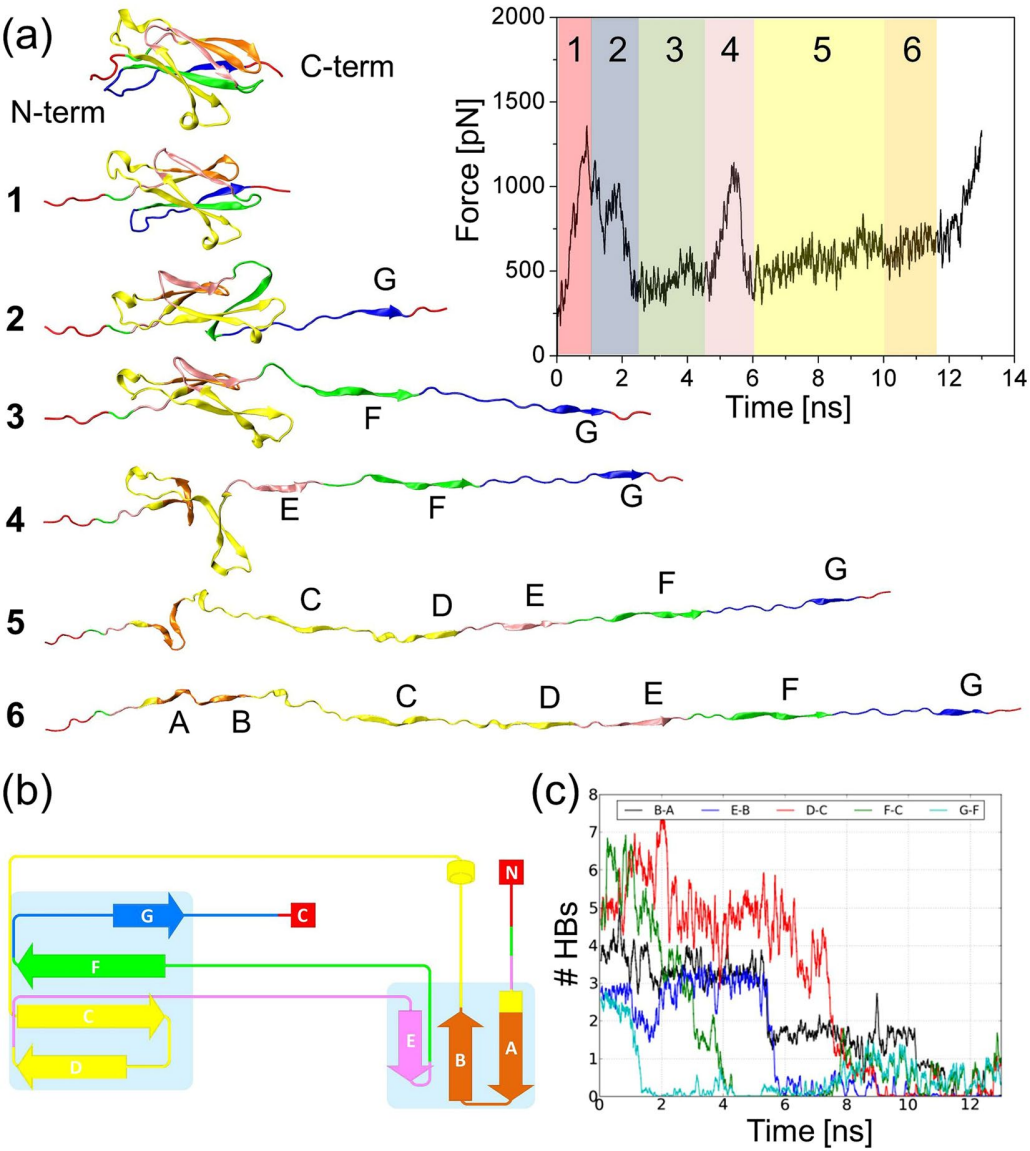
Gradual unfolding of the FnIII_3_ domain of CNTN4. Snapshots from simulations at various time points of the mechanical unfolding are shown. The accompanying time evolution of force is shown on the upper right. (**b**) Schematic structure of FnIII_3_ domain of CNTN4. Colors denote the sequence of unfolding events. (**c**) Change in the number hydrogen bonds traced during the course of stress-induced unfolding for 5 pairs of β-strands making hydrogen bonds in the folded state.

To characterize the differential mechanical response of each CNTN4 domain in the presence of buffering neighboring modules, ten 80 ns SMD runs were conducted for the full length protein and seven short (12–14-ns) SMD simulations were additionally performed for the individual modules. These are the first SMD simulations of the full-length contactin. Figure 5 displays the result from the force-induced unfolding of intact CNTN4. Panels a-b display the force profiles as a function of time (*top* curves) as well as the time evolution of the (disruption of) hydrogen bonds in the individual domains (*bottom* curves). The force generally increases with time, with several peaks indicating the barriers to unfolding or melting of connections in the tertiary structure in each module. Dotted lines (*top panels, inset*) indicate the time intervals associated with the response of individual FnIII domains (*black* - FnIII_1_, *red* - FnIII_2_, *green* - FnIII_3_, *blue* - FnIII_4_). Notably, the unfolding profile of the FnIII domains in the intact CNTN4 is very similar to that obtained for the isolated domain Fig. 6). This suggests that unfolding properties deduced from individual domains may be transferred to the whole protein nanomechanics interpretation, at least in the multidomain proteins composed of domains/modules connected by flexible linkers. Furthermore, the force-extension profiles of FnIII modules have a shared “fingerprint” specific to this family (see Fig. 6 for FnIII_3_).

The unfolding process of each FnIII domain can be divided into five parts based on current observations (Fig. 6a): I) pre-burst state, from ΔL = 0 to around 1 nm (0.5 ns), during which the domain maintains its β-sheet structure and the external force remains smaller than 1000 pN; II) main burst state, from 1 nm to around 3.5 nm (1.5 ns), when the secondary structure begins to break down; III) post-burst state completed with characteristic intermediate state, from 3.5 nm to 13.5 nm (5.5 ns), during which each individual FnIII domain unravels from the C-terminus and gradual dissolution of two F and G strands (see Fig. 6) can be observed; IV) post-burst state, recorded after the intermediate state, from 13.5 nm to around 30–32 nm (~13 ns), during which the elongation of an individual β-strand can be observed starting from breaking all connections between E and B strands (Fig. 6) and those interactions are also visible in AFM spectra of CNTN4 (Fig. 4a, *green arrow*); V) fully elongated (>30–32 nm), when the protein polypeptide chain achieves maximal length. FnIII domain is also present in other proteins such as fibronectin (FN)^43–45^. The FnIII_1_, FnIII_9_ and FnIII_10_ domains of FN reveal unfolding profiles similar to those of the FnIII domains of CNTN4, where the characteristic peak at ~13 nm originating from the disruption of hydrogen bonds between the E and B strands is also clearly distinguished^43,45^. However, there are also other members among FnIII domain family in which the force profile is different and the characteristic peak at ~13 nm is not observed, for example FnIII_7_ of FN. That may suggest FnIII domains, while sharing many features, may also retain protein-specific features. After the complete rupture of tertiary structures of FnIII modules, around ~50 ns, the first IgC2 hydrogen bond began to break (see Fig. 5).

During the course of simulations with the force applied to the C-terminus, two types of elongation processes were observed: (1) non-cooperative, when the unfolding pattern can be divided into the individual profiles of single FnIII domains and each module is elongated independently (Fig. 5a); then, the sequence of unfolding event is rather dependent upon the application point of the tension; and (2) partially cooperative, when some part of FnIII_2_ domain elongates at the same time as FnIII_3_ domain (Fig. 5b). This suggests that FnIII_2_ has a structure less mechanically resistant than FnIII_3_ module. That observation could be verified by the *MechStiff *^ 30^ comparison of each module (Fig. 5c). Results from mechanical stiffness calculations showed good agreement with the results for individual FnIII domain (FN) stretching. FnIII_3_ and FnIII_1_ gave rise to the largest proportion of high force constants (over 15 a.u.) consistent with their elevated intrinsic stiffness, whereas FnIII_2_ and FnIII_4_ exhibited the softest unfolding forces. Our experience suggests that this type of analysis can be successfully used for predicting the mechanical resistance of every structure before starting a SMD simulation. SMD indicates the maximal unfolding force for the individual IgC2 modules rank-ordered as: IgC2_3_ (1,625 ± 31 pN), IgC2_2_ (1,518 ± 114 pN) and IgC2_4_ (1,486 ± 52 pN), i.e., unfolding of these domains necessitates the application of more than 1,500 pN to break internal connections. The other modules of IgC2 have lower F_max_ values: IgC2_6_: 1,242 ± 177 pN, IgC2_5_: 1,301 ± 27 and IgC2_1_: 1,325 ± 93 pN. A detailed description of unfolding events in individual IgC2 domains has been published earlier.

When the tension is applied to the N-terminus, the unfolding pattern changes significantly (Fig. 5b). We note, in particular, the inversion in the order of individual modules that are sequentially unfolded. The general trend is the unfolding of the modules closest to the point of application of the perturbation (IgC2 domains in this case, as opposed to FnIII domains in the opposite case of a tension exerted to the C-terminus). And even among the different IgC2 domains those closest to the perturbation site exhibit the earliest response and there is a gradual propagation of unfolding away from the application point. Considerable increase in force occurs until the force reaches 3.5 nN at 35 ns when all IgC2 modules and a part of (the first neighboring) FnIII_1_ are extended. The cooperative melting of the secondary structure, at the same time in IgC2_1_ and IgC2_2_ modules, takes 10 ns. A similar structural response could be observed in IgC2_3_ and IgC2_5_ domains. In the simulation of the whole CNTN4 it is hard to discern the mechanical strength of a single IgC2 type domain. Instead of showing pronounced unfolding peaks for FnIII domains and increase in force as a function of time, our trajectory shows a relatively stable plateau and a few slightly increased force peaks with visible positions. This analysis clearly demonstrates that there may be multiple unfolding pathways due to the complexity of the structure as well as non-equilibrium properties of the simulated process, and the site of application of the stress tends to induce early unfolding events at its close vicinity.

## Conclusion

CNTN4 plays diverse functions. It plays a crucial role in the formation and maintenance of mice functional odor map in the olfactory bulb^47^. There is increasing evidence that mutations affecting human CNTN4 structure may be associated with 3p deletion syndrome^5^ and/or autism spectrum disorders (ASD)^9,48^. Recent studies indicate that the absence of CNTN4 or its binding partner APP^49^ in neuronal cells has an impact on target-specific axon arborization, which in turn affects functional development of a visual pathway^50^. Our study provides first insights into the mechanical behavior of this important member of the CNTN family of CAMs.

The mechanical response of single or multiple domain proteins to uniaxial tension has been computationally investigated in a number of insightful studies^27–29,44–46^. Here we report for the first time all-atom SMD unfolding simulations of a complete hetero-modular protein, over one thousand amino acids long, along with elastic-network-model analysis of the mechanical stiffness of the intact protein. Such domains are abundant in numerous proteins, and the combined smAFM and SMD analysis emerges as a useful approach for delineating the nanomechanics of protein domains.

Our study brings new results in the field of nanomechanics of CAMs composed of mixed numbers of Ig/FnIII type protein modules. In our smFS data we noticed patterns that may be attributed to the extension of the horseshoe conformation, a bent shape adopted by numerous CAM proteins (Fig. 4d). It means that a smFS experiment can help probe, not only the unfolding force and extension of individual modules, but the specific arrangements of modular domains. The change from horseshoe to extended form comes with a cost. Those attractive interactions between IgC2_1_-IgC2_4_ and IgC2_2_-IgC2_3_ in the horseshoe conformer require ~20 pN force to be broken. We expect that this type of “event” will be observed in smFS experiments for other cell-adhesion proteins. However, a firm proof that this is the only explanation of smAFM spectra observed here requires studies of engineered CNTN4 variants, which is beyond the scope of the present work.

In our study, we collected an ensemble of spectra (>2,800 of CNTN4 domains unfolding events) and analyzed it in great detail. CNTN4 unfolding profile was characterized by three unfolding lengths: ~31 nm - assigned to the FnIII domains, and ~10 nm and ~18 nm linked with IgC2 domains restrained by disulfide bonds (Figs 1 and 2). Those values are in good agreement with computational results for fully stretched single FnIII (29–33 nm) and IgC2 (10–18 nm) modules. Due to the variability in the sizes and sequences of the domains of the same type, we observed two broad types of FnIII domains responding to two different unfolding forces, ~80 pN and ~145 pN. Both types of mechanical response have been detected^34^ in titin as well: FnIII_10, 13_ ~70–90 pN and FnIII_12_ ~125 pN. Our data further confirm the presence of intermediate states in those modules (Fig. 4a) which can be discerned during the course of mechanical unfolding. These observations correlate well with SMD results suggesting that the intermediate originates from the disruption of interactions between the E and B strands (~13 nm, Fig. 5). Such events have been observed in computational stretching of FnIII domains. Here we presented experimental data which confirm that for the same domain there might be alternative unfolding paths, namely with and without the intermediate state, depending on the site of application of perturbation/stress that induces unfolding (Fig. 4a). Furthermore, we present here for the first time that some of the IgC2 domains in CNTNs reach their maximal deformation with forces that are ~2 times weaker than those observed till now in other proteins^34,36,37^, although their overall extension falls short of that of FnIII domains due to disulfide bonds that retain partial structure. Our observations may help build biological models of CAMs and their functions in neurons and/or synaptic clefts. Furthermore, the mechanical signals provided by AFM FS spectra, complemented by computational analyses, may help determine the overall shape of multidomain proteins adsorbed on a surface and their time-dependent reconfiguration, which may be exploited in further studies of nanostructures.

## Materials and Methods

### Atomic Force Microscopy

Human recombinant CNTN4 was purchased from R&D system (Accession # Q8IWV2). This protein contains the human CD33 signal peptide (Met1 - Ala16), and a 10 amino-acid stretch of histidines at C-terminus, in addition to CNTN4 residues Asp19 - Gly1001. The smFS measurements were performed using JPK NanoWizard 3 AFM. Olympus OMCL-TR400PSA-HW cantilevers with normal spring constant 0.02 N/m were calibrated using the thermal tune method^51^ before each experiment. Thermo Scientific Microscope Cover Glass was treated with Harrick Plasma cleaner PDC-32G. Air pressure of (1000 ± 50) mTorr at low (30 sec) intensity was used. The protein was suspended in pure PBS buffer without any accompanying proteins added at a concentration of 50 µg/ml and deposited on the glass plate directly after plasma treatment. Protein was attached to the glass substrate via nonspecific binding and no chemical modifications were involved in the sample preparation. In our smAFM experiment, the protein sample is placed on a glass coverslip which is attached to the piezoelectric stage; single molecules of CNTN are picked up randomly by adsorption to the AFM tip and subjected to uniaxial tension by forces applied to their N- and C-termini. Time of 15 min of incubation was kept in each experiment. The typical pulling speed was about 0.5 µm/s whereas unfolding traces were collected using JPK dedicated software for automatic sampling in a Force Spectroscopy mode. A gird with sampling points (around 100) and sampling frequency (1–5 times per point) were used by the robot-enhanced JPK AFM during the FS scanning mode. We collected over 104,000 curves from which we selected over 500 good quality traces showing at least four clear maxima. Data analysis was made using JPK Data Processing program and home-made scripts. The worm-like chain (WLC) model^52^ from JPK program was used to evaluate the length increments accompanying the unfolding events.

### Steered Molecular Dynamics

All-atom SMD simulations were performed for human CNTN4 (H24-A995) using the NAMD^53^ software and the CHARMM27 force field^54^. Due to the absence of a high-resolution structure of CNTN4, a homology model has been created using Swiss Model^55^ and home-made scripts, using as template other IgC2 and FnIII domains from different forms of CNTNs and CAMs available in the PDB. The SMD simulation time was over 1 µs. In order to reduce the CPU power, the number of water molecules in the solvation shell was gradually reduced and the stability of the protein was verified. The number of TIP3P waters varied from 218,500 (0.7 nm water box) to 9,500 in the thinnest water layer (0.3 nm), and further tests with implicit solvent SMD were carried out (Supplementary Material). A cutoff of 12 Å for non-bonded interactions was applied. Langevin dynamics and the Langevin piston algorithm were used to maintain the temperature at 300 K and the pressure at 1 atm^56^. We adopted the following protocol: 0.2 ns of water equilibration, 10,000 steps of minimization, 0.35 ns of heating from 0 to 300 K, and 0.15 ns equilibration of the whole system before initiating each SMD run. Constant velocity SMD scheme was used to stretch the CNTN structure along its N-to-C vector (also called *end-to-end vector*) at a constant speed of 0.025 Å/ps. The N-to-C vector connects the C_α_-atoms of the N- and C-terminal residues located at positions obtained after water equilibration. In ten simulations the C-terminus of the structure was fixed and a harmonic force with a spring constant of 278 pN/Å was applied to the N-terminus. In one trajectory, the direction of pulling was reversed. We have performed 10 independent SMD runs, each 80 ns long. VMD^57^, *MechStiff* module^30^ of *ProDy*^58^, and home-made scripts were used for visualizing and analyzing trajectories. *MechStiff* module was used to evaluate the effective resistance of residues to deformation i.e. effective spring constants which were further counted for individual domain. Additionally, the unfolding trajectories of the individual domains were simulated to investigate the influence of neighboring module ballast on the unfolding pathway of a given unit (Supplementary Material).

### Data Availability

The datasets generated during the current study are available here: http://www.fizyka.umk.pl/~karolamik/data/.

## Electronic supplementary material

Supplementary material
