# Author Correction: Nanomechanics of multidomain neuronal cell adhesion protein contactin revealed by single molecule AFM and SMD

**DOI:** 10.1038/s41598-018-21746-7

**Published:** 2018-03-06

**Authors:** Karolina Mikulska-Ruminska, Andrej J. Kulik, Carine Benadiba, Ivet Bahar, Giovanni Dietler, Wieslaw Nowak

**Affiliations:** 1 Laboratory of Physics of Living Matter, Ecole Polytechnique Fédérale de Lausanne (EPFL), CH-1015 Lausanne, Switzerland; 2 Institute of Physics, Faculty of Physics, Astronomy and Applied Informatics, Nicolaus Copernicus University, Grudziadzka 5, 87-100 Torun, Poland; 3 Department of Computational and Systems Biology, School of Medicine, University of Pittsburgh, 3501 Fifth Ave, Biomedical Science Tower 3, Pittsburgh, PA 15213 USA

Correction to: *Scientific Reports* 10.1038/s41598-017-09482-w, published online 18 August 2017

The original version of this Article contained typographical errors in the spelling of the authors “Karolina Mikulska-Ruminska, Andrej J. Kulik, Carine Benadiba, Ivet Bahar, Giovanni Dietler & Wieslaw Nowak” which were incorrectly given as “K. Mikulska-Ruminska, A. J. Kulik, C. Benadiba, I. Bahar, G. Dietler & W. Nowak”. This has now been corrected in the PDF and HTML versions of the Article, and in the Supplementary Information file.

