# Supplementary material for: Nanomechanics of multidomain neuronal cell adhesion protein contactin revealed by single molecule AFM and SMD
Source: Sci Rep. 2017 Aug 18;7:8852. doi: 10.1038/s41598-017-09482-w (PMC5562865; doi:10.1038/s41598-017-09482-w)
Supplement: Supplementary file 1 — Supplementary material [file 41598_2017_9482_MOESM1_ESM.pdf]

Supplementary materials for

Nanomechanics of multidomain neuronal cell adhesion protein contactin revealed by single molecule AFM and SMD

Karolina Mikulska-Ruminska[1, 2, 3,*], Andrej J. Kulik[1], Carine Benadiba[1], Ivet Bahar[3,*], Giovanni Dietler[1] and Wieslaw Nowak[2]

[1] Laboratory of Physics of Living Matter, Ecole Polytechnique Fédérale de Lausanne (EPFL), CH-1015 Lausanne, Switzerland, [2] Institute of Physics, Faculty of Physics, Astronomy and Applied Informatics, Nicolaus Copernicus University, Grudziadzka 5, 87-100 Torun, Poland, [3] Department of Computational and Systems Biology, School of Medicine, University of Pittsburgh, 3501 Fifth Ave, Biomedical Science Tower 3, Pittsburgh, PA 15213, USA.

* Corresponding authors: karolami@pitt.edu (KMR), bahar@pitt.edu (IB)

Estimation of CNTN4 modules lengths

Table S1. Structural arrangement of CNTN4 domains. The number of residues for each module includes disulfide bridges (SS) in IgC2 modules (No. SS-SS). Estimated N-C final length of each module is based on the number of not restrained residues multiply by the peptide length (0.36 and 0.4 nm/residue). ΔL is calculated by subtracting estimated N-C final length and initial length of N-C terminal (L0). Table includes the contribution from linkers.

	Number of residues [aa]			Estimated N-C final length of domain [nm]					
Module	No. N-SS	No. SSSS	No. SS-C	.36 nm/residue			0.4 nm/residue		
				N-SS	SS-C	N-SS + SS-C	N-SS	SS-C	N-SS + SS-C
**IgC2$_1$**	18	**51**	17	6.48	6.12	**12.6**	7.2	6.8	**14**
**IgC2$_2$**	22	**51**	13	7.92	4.68	**12.6**	8.8	5.2	**14**
**IgC2$_3$**	22	**49**	16	7.92	5.76	**13.68**	8.8	6.4	**15.2**
**IgC2$_4$**	21	**48**	16	7.56	5.76	**13.32**	8.4	6.4	**14.8**
**IgC2$_5$**	23	**49**	16	8.28	5.76	**14.04**	9.2	6.4	**15.6**
**IgC2$_6$**	22	**58**	10	7.92	3.6	**11.52**	8.8	4	**12.8**
**FnIII$_1$**		99				**35.64**		9.6	**39.6**
**FnIII$_2$**		98				**35.28**		9.2	**39.2**
**FnIII$_3$**		96				**34.56**		8.4	**38.4**
**FnIII$_4$**		96				**34.56**		8.4	**38.4**

	Number of residues [aa]				ΔL [nm]		Estimated contribution for linkers between domains [nm]		
Module	No. N-SS	No. SSSS	No. SS-C	*L0 [nm]	ΔL (0.36 nm)	ΔL (0.4 nm)	No. AA [aa]	0.36 nm/aa	0.4 nm/aa
**IgC2$_1$**	18	**51**	17	3.8	**8.8**	**10.2**	4	1.44	1.6
**IgC2$_2$**	22	**51**	13	4.1	**8.5**	**9.9**	17	6.12	6.8
**IgC2$_3$**	22	**49**	16	4.3	**9.38**	**10.9**	4	1.44	1.6

**IgC2$_4$**	21	**48**	16	3.7	**9.62**	**11.1**	5	1.8	2
**IgC2$_5$**	23	**49**	16	3	**11.04**	**12.6**	3	1.08	1.2
**IgC2$_6$**	22	**58**	10	2.4	**9.12**	**10.4**	12	4.32	4.8
**FnIII$_1$**		99		3.5	**32.14**	**36.1**	4	1.44	1.6
**FnIII$_2$**		98		3.5	**31.78**	**35.7**	4	1.44	1.6
**FnIII$_3$**		96		2.5	**32.06**	**35.9**	0	0	0
**FnIII$_4$**		96		2.4	**32.16**	**36**			

** Initial N-C terminal length of the domain measured from computational model.*

Calculation in the Tab. S1 was carried out by assuming that the length of a single stretch of amino acids (aa) is 0.36 nm or 0.40 nm, which corresponds to the separation of the CA atoms of two adjacent aa in the extended conformation. For example for FnIII$_4$ domain we calculated 96 aa × 0.36 nm/aa = 34.56 nm. We subtracted the diameter of the folded domain (L0 = 2.4 nm) from this value, giving a theoretical contour length of 32.16 nm, which is very close to the experimental one. In similar way all values were calculated for peptide length 0.4 nm/aa. IgC2 module residues were split in to three regions: from C-terminal to disulfide bridge (N-SS), between disulfide bridges (SS-SS), and from disulfide bridge to N-terminal part of the module (SS-C).

Water shell simulations

In **Fig. S1** three SMD force-time (~extension) profiles of CNTN4 immersed in a water shell stretched at a constant speed of 0.025Å/ps. The simulations were stopped after 80 ns when the protein was completely unfolded. In **Fig. S1a,c** data for shells of different size of the water box i.e. 0.7 nm and 0.3 nm (219,000 and 166,000 atoms) are presented. The external force was applied to the C-terminus while the N-terminus was restrained. The profiles show constant increase of force with some characteristic peaks up to the value of 3.5 nN. At the beginning mainly non-cooperative unfolding of FnIII domains can be observed in the following order FnIII$_4$, FnIII$_3$, FnIII$_2$ and FnIII$_1$ (see **Fig. S1a,c**). During the unfolding of the FnIII$_4$ module two characteristic maxima appear on the force profile: the first at ~1 ns and the second at ~5 ns. Around 5 ns the force is high enough to break hydrogen bonds in the module; it takes 1 ns. This indicates an intermediate state in the FnIII$_4$ domain. FnIII$_3$ stretching also reveals a high maximum at the first unfolding step. The unfolding process of the other FnIII modules is characterized by slightly lower incensement in force. All FnIII modules are stretched in ~50 ns of simulations. After complete rupture of the tertiary structures of FnIII modules the first IgC2 hydrogen bonds start breaking. The sequence of unfolding domains dependents on the application point of external force. Starting from the weakest domain, the stretching process follows the structural order: IgC2$_6$, IgC2$_5$, IgC2$_4$, IgC2$_1$, IgC2$_3$, IgC2$_2$ (**Fig. S1 a,c**). IgC2$_1$ is the only exception where complete melting of the secondary structure comes sooner than expected.

We performed SMD simulation in which the external force is applied to the N-terminus of CNTN4 as well. The unfolding pattern has changed significantly (see **Fig. S1b**).

It is expected since the SMD unfolding protocol by its very nature is a non-equilibrium process. Substantial increase of force occurs until the force reaches 3.5 nN at 35 ns when all IgC2 modules and a part of FnIII$_1$ are elongated. Cooperative melting of the secondary structure of IgC2$_1$ and IgC2$_2$ modulus occurs at the same time and it takes about 10 ns. A similar structural response can be observed in IgC2$_3$ and IgC2$_5$ domains. The strongest Ig domains were IgC2$_4$ and IgC2$_6$. Next, in contrast to pronounced unfolding force peaks for FnIII domains as in previous simulations (**Fig . S1a,c**), the profile shows continuous stable force plateau and a few distinct small force peaks. The further unfolding trajectory reveals a rather continuous structural change of FnIII domains upon stretching of FnIII$_1$, FnIII$_2$, FnIII$_3$, FnIII$_4$ respectively.

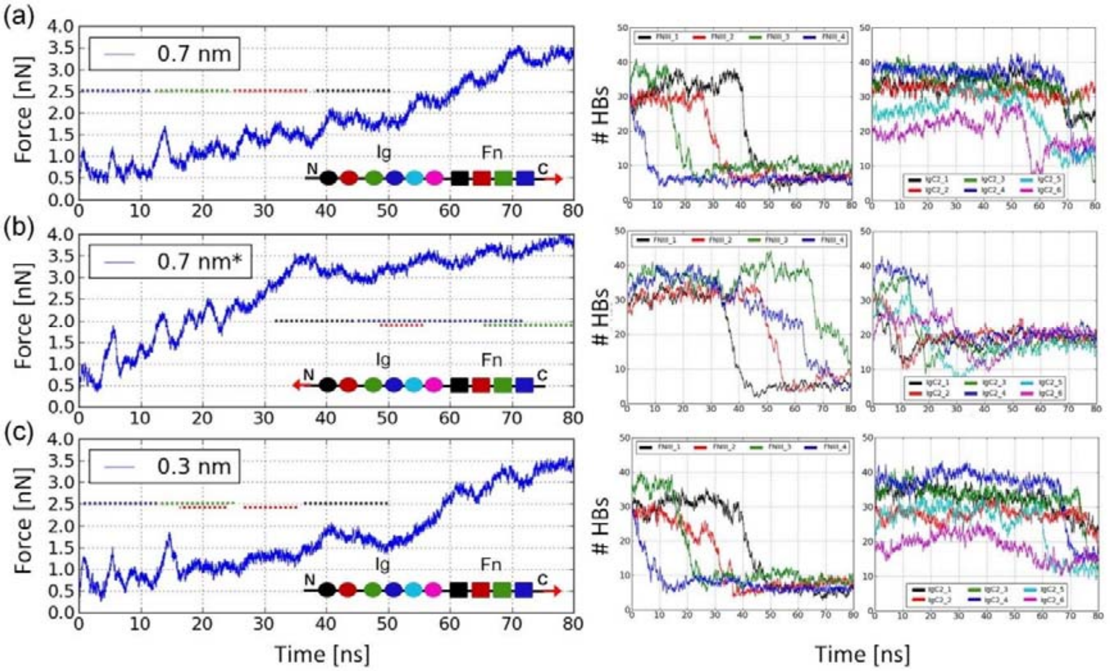

Figure S1. Water shell SMD simulations of CNTN4 with: (a) 0.7 nm, (b) 0.7 nm (opposite direction of pulling) and (c) 0.3 nm size of water layer. For each simulation force-time profile is shown with a modular structure of CNTN4 showing the direction of pulling and the number of hydrogen bonds (# HBs) registered during the unfolding process of each domain. The colors encode the corresponding CNTN4 modules. The dotted lines indicate the place of the profile where the secondary structure melting of the marked FnIII domain occurs.

Water layer simulations

Four SMD simulations with different dimension of water layer hydrating CNTN4 protein have been performed. Fig. S2 shows the unfolding traces with N-to-C pulling vector of protein in 1.2 nm, 0.5 nm and 0.3 nm thick TIP3P solvent coats. The water layer system is characterized by the significant smaller water number than in water box system (90,000, 31,000, and 25,000 atoms). The sequence of unfolding events in this simplified water system is quite similar to the water box simulations. However, the number of water molecules in the model may have some impact on the unfolding scenario of IgC2 domains because after stretching of $IgC2_6$ and $IgC2_5$ at about 60 ns we cannot observe other major unfolding among Ig modules (see Fig. S2).

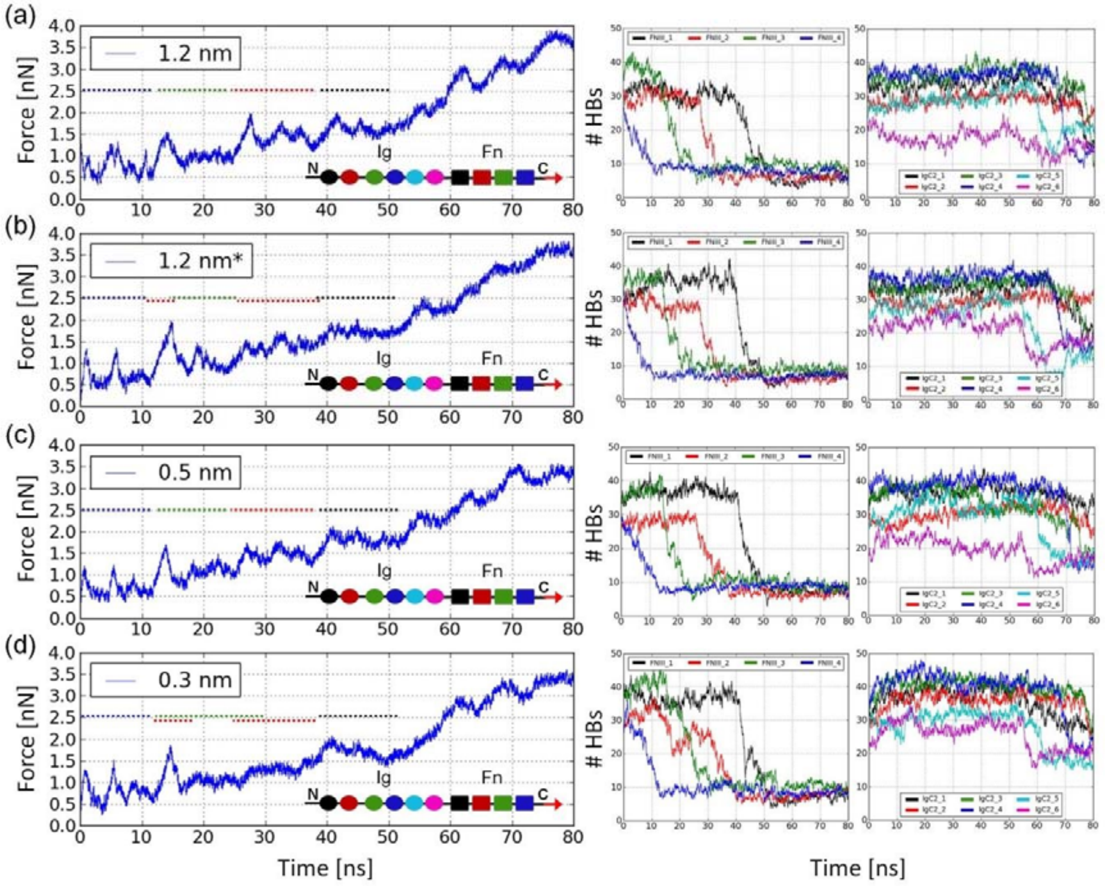

Figure S2. Water layer SMD simulations of CNTN4 with: (a) 1.2 nm, (b) 1.2 nm (c) 0.5 nm (d) 0.3 nm water shells. For each simulation force-time profile (SMD single molecule force spectrum) is shown together with a modular structure of CNTN4, indicated the direction of pulling and the number of hydrogen bonds (# HBs) registered during the unfolding. The colors encode the corresponding CNTN4 modules. The dotted lines mark places of the profiles where the secondary structure melting of a particular FnIII domain occurs.

Implicit solvent simulations

To reduce large computational cost and some practical limitations of fully atomistic simulations an implicit solvent approach has been used to simulate the unfolding of CNTN4. The unfolding pathways for different dielectric constant, respectively of 1, 18 and 81, are presented in **Fig. S3**. Force profiles with dielectric constant 18 and 81 show a small number of interactions in individual modules during the unfolding process (see **Fig. S3b,c**). Both curves have a rather low force plateau with low force peaks just above the noise level. Nevertheless, characteristic pattern for individual FnIII domain and the unfolding scenario could be identified and are marked by dotted lines in **Fig. S3b,c**. The number of hydrogen bonds calculated with standard distance/angle parameters is extremely low, less than 10 in each module. Therefore, it suggests low free energy barriers in the mechanical unfolding pathway of CNTN4 when high dielectric constant is used in the modeling. At 80-ns the level of force reaches 2 nN which is nearly two times smaller than that observed in explicit solvent simulations.

The unfolding profile for $\epsilon = 1$ significantly changed the character (**Fig. S3a**) in comparison to epsilon 18 or 81. The number of hydrogen bonds radically increases. Therefore, large fluctuations of forces are prevailing for each individual module. The unfolding scenario is different than in the explicit and other implicit solvent simulations. These data show that the value of the dielectric constant significantly changes the force-time profile of CNTN4 elongation.

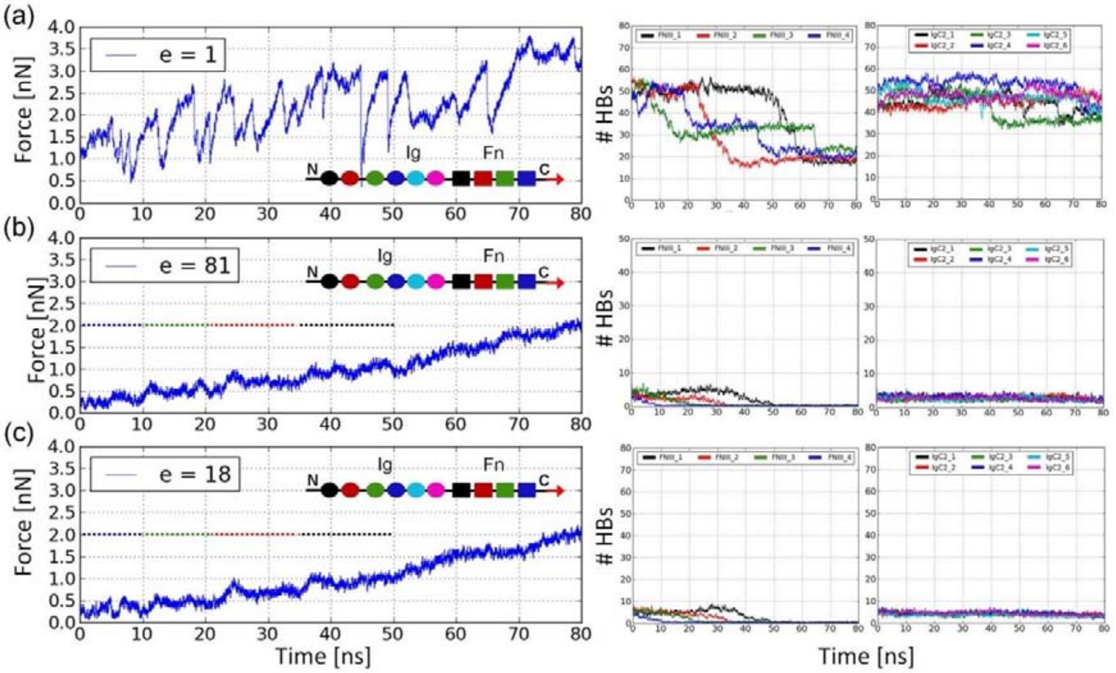

Figure S3. Force-time profiles and the number of hydrogen bonds in CNTN4 modules during implicit solvent SMD simulations with the dielectric constant ϵ: (a) 1 (b) 81 (c) 18. For each simulation force-time pattern is shown with modular structure of CNTN4 viewing direction of pulling and the number of hydrogen bonds (# HBs) registered during the unfolding. The colors encode the corresponding CNTN4 modules. The dotted lines mark places of the profiles where the secondary structure melting of a particular FnIII domain occurs.

The unfolding of compact CNTN4 reveals two types of elongation process: I) non cooperative, when the unfolding pattern can be divided into the individual profiles of single FnIII domains cause each module is elongated independently (see **Fig. S1a, S2a, S2c, S3b-c**). Moreover, the sequence of unfolding event is rather dependent upon the direction of pulling and the point of application of force vector; II) partially cooperative, when some part of $FnIII_2$ domain elongates at the same time as $FnIII_3$ domain (**Fig. S1c, S2b, S2d**).
